# Association Between Waiting Time from Diagnosis to Treatment and Survival in Patients with Curable Gastric Cancer: A Population-Based Study in the Netherlands

**DOI:** 10.1245/s10434-017-5820-8

**Published:** 2017-03-28

**Authors:** H. J. F. Brenkman, E. Visser, P. S. N. van Rossum, S. Siesling, R. van Hillegersberg, J. P. Ruurda

**Affiliations:** 1 Department of Surgery, University Medical Center Utrecht, Utrecht, The Netherlands; 2 Department of Radiation Oncology, University Medical Center Utrecht, Utrecht, The Netherlands; 3 Department of Research, Netherlands Comprehensive Cancer Organisation, Utrecht, The Netherlands; 4 Department of Health Technology and Services Research, MIRA Institute for Biomedical Technology and Technical Medicine, University of Twente, Enschede, The Netherlands

## Abstract

**Background:**

In the Netherlands, a maximum waiting time from diagnosis to treatment (WT) of 5 weeks is recommended for curative cancer treatment. This study aimed to evaluate the association between WT and overall survival (OS) in patients undergoing gastrectomy for cancer.

**Methods:**

This nationwide study included data from patients diagnosed with curable gastric adenocarcinoma between 2005 and 2014 from the Netherlands Cancer Registry. Patients were divided into two groups: patients who received neoadjuvant therapy followed by gastrectomy, or patients who underwent gastrectomy as primary surgery. WT was analyzed as a categorical (≤5 weeks [Reference], 5–8 weeks, >8 weeks) and as a discrete variable. Multivariable Cox regression analysis was used to assess the influence of WT on OS.

**Results:**

Among 3778 patients, 1701 received neoadjuvant chemotherapy followed by gastrectomy, and 2077 underwent primary gastrectomy. In the neoadjuvant group, median WT to neoadjuvant treatment was 4.6 weeks (interquartile range [IQR] 3.4–6.0), and median OS was 32 months. In the surgery group, median WT to surgery was 6.0 weeks (IQR 4.3–8.4), and median OS was 25 months. For both groups, WT did not influence OS (neoadjuvant: 5–8 weeks, hazard ratio [HR] 0.82, *p* = 0.068; >8 weeks, HR 0.85, *p* = 0.354; each additional week WT, HR 0.96, *p* = 0.078; surgery: 5–8 weeks, HR 0.91, *p* = 0.175; >8 weeks, HR 0.92, *p* = 0.314; each additional week WT, HR 0.99, *p* = 0.264).

**Conclusions:**

Longer WT until the start of curative treatment for gastric cancer is not associated with worse OS. These results could help to put WT into perspective as indicator of quality of care and reassure patients with gastric cancer.

Gastric cancer is the fifth most common type of cancer worldwide.^1^ In the Netherlands, only a third of patients with gastric cancer qualify for curative treatment, which consists of surgical resection with D2 lymphadenectomy with or without perioperative chemotherapy.^2^,^3^ Unfortunately, the 5-year overall survival (OS) after curative treatment remains poor (35–45%).^3^–^5^

The interval from diagnosis to treatment (waiting time, WT) is considered to be an important quality indicator for cancer care because it negatively influences patients’ quality of life, results in psychologic distress, and has been demonstrated to be associated with oncologic outcomes in various cancers.^6^–^8^ Hence, in the Netherlands, WT to the start of curative treatment for gastric cancer is an important quality indicator as recommended by the Dutch Hospitals Association, the Dutch Federation of University Medical Centres, the Ministry of Health, Welfare and Sport, and the Association of Surgical Oncologists.^6^,^9^,^10^ However, the biology and behavior of tumors vary, indicating that the impact of WT for each type of cancer may differ. Therefore, there is a need for cancer-specific recommendations.

Only one study has reported on this topic for gastric cancer specifically.^11^ This study was performed in the Asian population, which differs significantly from the Western population and generally does not undergo neoadjuvant treatment.^11^,^12^ The aim of the current study was to evaluate the association between WT and OS in a large Western population-based cohort of patients undergoing curative gastrectomy with or without perioperative chemotherapy for cancer. It was hypothesized that longer WTs are associated with worse OS as a result of disease progression.

## Materials and Methods

### Study Design

This population-based cohort study was conducted with data from the Netherlands Cancer Registry (NCR). In the Netherlands, a population of 17 million inhabitants, all newly diagnosed cancers are registered in the NCR, which is hosted by the Netherlands Comprehensive Cancer Organisation (IKNL). The main source of notification is the National Automated Pathology Archive, which sends weekly notifications of all cancer cases. Furthermore, yearly, the National Registry of Hospital Discharge Diagnoses is linked to the NCR to obtain clinical cancer diagnosis only. On a daily basis, trained data managers register data from hospital records within all Dutch hospitals using the NCR’s registration and coding manual. The privacy committee of the NCR approved this study.

### Patient Population

In this study, all patients diagnosed with curable gastric adenocarcinoma (cT1–4a, N0–3, M0) in the period 2005–2014 were selected from the NCR. Exclusion criteria consisted of neoadjuvant therapy other than chemotherapy and emergency gastrectomy (gastrectomy ≤14 days after diagnosis or start of neoadjuvant treatment). Patients who were diagnosed with curable gastric cancer (cTNM) but who underwent a palliative resection (pTNM) were deliberately included in this study, as this might reflect disease progression during WT.

Disease was staged, and patients underwent treatment according to the nationwide guidelines for gastric cancer.^2^ Staging consisted of a gastroscopy and a computed tomographic scan in all patients, whereas endoscopic ultrasound and diagnostic laparoscopy were not routinely performed. Before 2006, the standard of treatment consisted of gastrectomy only, and after that, an increasing number of patients underwent perioperative chemotherapy.^3^ Surgery consisted of a distal or total gastrectomy, depending on the possibility to achieve an adequate proximal resection margin (≥6 cm), along with a D2 lymphadenectomy (without station 10 dissection and without pancreaticosplenectomy).^13^,^14^ The resected specimens were reviewed by pathologists and presented according to the American Joint Committee on Cancer tumor, node, metastasis classification system (TNM) staging system (7th edition).

### Statistical Analysis and Outcome Measures

All included patients were divided into two groups: a neoadjuvant group and a surgery group. Patients in the neoadjuvant group received neoadjuvant chemotherapy (as part of perioperative chemotherapy) followed by gastrectomy, whereas patients in the surgery group underwent gastrectomy only. The WT was calculated in weeks and was defined as the interval between the date of diagnosis and the start date of neoadjuvant chemotherapy for the neoadjuvant group, and as the interval between the date of diagnosis and the date of gastrectomy for the surgery group. The date of diagnosis was used as the date of the first gastrointestinal endoscopy, on which the diagnosis of gastric cancer had been established by histology from biopsy samples. The WT constitutes a combination of time needed to confirm the diagnosis of gastric cancer, referral time, staging, pretherapeutic assessment, and actual WT for treatment.^2^ WT was grouped into three categories of ≤5 weeks, 5–8 weeks, and >8 weeks on the basis of national recommendations and previous studies.^6^,^15^,^16^ In both groups, a subgroup was made of patients who underwent a curative gastrectomy (pT1–4aN + M0) and palliative gastrectomy (detected unresectable tumor [pT4b] and/or metastatic disease [M1] intraoperatively). Missing baseline data were considered at random and handled using multiple imputation with the iterative Markov chain Monte Carlo methods (20 iterations).

To assess the association between WT and tumor progression, the median WT was compared between the curative and palliative treated patients by the Mann–Whitney *U* test. To assess the distribution of all baseline, surgical, and histopathologic characteristics in the different WT groups (≤5, 5–8, >8 weeks), all baseline, surgical, and histopathologic characteristics were compared among the three groups of WT. Categorical variables were analyzed by the Chi square test, and continuous variables were compared by the parametric ANOVA test.

To assess the association between WT and OS, univariable and multivariable analyses by means of Cox proportional hazard models were used, providing hazard ratios (HRs) with 95% confidence intervals. WT was analyzed as a categorical variable and as a discrete variable. All baseline variables and WT were entered in a multivariable analysis. Sensitivity analyses were performed for the following subgroups: cT1–2 versus cT3–4, good to moderate versus poor to undifferentiated tumors, and cN0 versus cN+. OS was defined as the time from the start date of treatment to the date of death from any cause or to the date of last follow-up (December 2015). A *p* value of <0.05 was considered statistically significant. All statistical analyses were performed by IBM SPSS 21 for Windows.

## Results

### Study Population

The NCR selected data from 4088 patients diagnosed with curable gastric adenocarcinoma (2005–2014). A total of 310 patients were excluded because of neoadjuvant therapy other than chemotherapy (*n* = 30) and emergency gastrectomy (*n* = 280). Of the remaining 3778 patients, 1701 received neoadjuvant chemotherapy followed by gastrectomy, and the other 2077 patients underwent primary gastrectomy.

Baseline characteristics of the neoadjuvant group are presented in Table 1. The mean age was 62.6 years, most of the patients were male (*n* = 1064, 63%), and most were clinically staged as having cT1–2 (*n* = 483, 55%) and cN0 (*n* = 951, 65%) disease. The majority underwent a distal gastrectomy (*n* = 924, 54%) and an open surgical approach (*n* = 1447, 86%). A curative gastrectomy was performed in 1544 patients (92%). The other 127 patients (8%) received a palliative resection (pT4b or pM1 intraoperatively).

**Table 1:** Baseline, surgical, and histopathologic characteristics of 1701 patients treated with neoadjuvant chemotherapy followed by gastrectomy for cancer

Characteristic	All (*N* = 1701)		<5 weeks (*n* = 895)		5–8 weeks (*n* = 451)		>8 weeks (*n* = 140)		*p*
	*n*	%	*n*	(%)	*n*	(%)	*n*	(%)	
Baseline characteristics									
Age, year, mean (±SD)	62.6	(±10.6)	62.4	(10.7)	63.2	(10.5)	62.4	(9.0)	0.386
Gender									0.479
Male	1064	(63)	548	(59)	287	(31)	92	(10)	
Female	637	(37)	347	(62)	164	(29)	48	(9)	
Malignancy history									0.934
No	1546	(91)	814	(60)	409	(30)	126	(9)	
Yes	155	(9)	81	(59)	42	(31)	14	(10)	
Tumor differentiation									0.163
Good to moderate	202	(20)	110	(21)	44	(17)	19	(26)	
Poor to undifferentiated	798	(80)	403	(79)	217	(83)	55	(74)	
Missing	701		382		190		66		
cT stage									0.399
T1	44	(5)	22	(55)	12	(30)	6	(15)	
T2	439	(50)	235	(56)	138	(33)	45	(11)	
T3	304	(35)	158	(55)	97	(34)	30	(11)	
T4a	88	(10)	33	(45)	26	(36)	14	(19)	
Tx	826		447		178		45		
cN stage									0.466
N0	951	(65)	504	(58)	277	(32)	82	(10)	
N+	511	(35)	285	(62)	134	(29)	42	(9)	
Nx	239		106		40		16		
Year of diagnosis									0.268
2006–2008	356	(21)	120	(59)	57	(28)	28	(14)	
2009–2011	616	(36)	339	(61)	170	(30)	49	(9)	
2012–2014	729	(43)	436	(60)	224	(31)	63	(9)	
Referral status									<0.001
Diagnosis in treatment hospital	1009	(69)	536	(53)	233	(23)	64	(6)	
Diagnosis in other hospital	445	(31)	211	(47)	144	(32)	53	(12)	
Missing	247		148		74		23		
Treatment and histopathologic characteristics									
Surgical type									0.687
Distal gastrectomy	924	(54)	487	(61)	236	(30)	77	(10)	
Total gastrectomy	749	(44)	394	(59)	211	(32)	62	(9)	
Multiorgan surgery	28	(2)	14	(74)	4	(21)	1	(5)	
Surgical approach									0.679
Open	1447	(86)	742	(60)	373	(30)	119	(10)	
Laparoscopic	230	(14)	141	(62)	69	(30)	18	(8)	
Missing	24		12		9		3		
Radicality									0.782
R0	1405	(86)	738	(60)	368	(30)	116	(9)	
R1–2	239	(15)	129	(61)	66	(31)	17	(8)	
Missing	57		28		17		7		
ypT stage									<0.001
T0	112	(7)	56	(57)	37	(37)	6	(6)	
T1	236	(14)	96	(48)	69	(34)	36	(18)	
T2	256	(15)	142	(62)	67	(29)	20	(9)	
T3	700	(42)	385	(62)	186	(30)	48	(8)	
T4a	309	(19)	162	(62)	73	(28)	25	(10)	
T4b	57	(3)	39	(74)	11	(21)	3	(6)	
Tx	31		15		8		2		
ypN stage									0.036
N0	805	(47)	387	(55)	234	(33)	80	(11)	
N1	338	(20)	193	(67)	78	(27)	19	(7)	
N2	274	(16)	147	(63)	64	(28)	21	(9)	
N3	282	(17)	168	(64)	75	(29)	20	(8)	
Nx	2								
ypM stage									0.357
M0	1625	(96)	885	(61)	438	(30)	135	(9)	
M1	76	(5)	40	(69)	13	(22)	5	(9)	
Curative intent									0.112
Yes	1544	(92)	807	(57)	419	(30)	188	(13)	
No	127	(8)	74	(62)	24	(20)	21	(18)	
Missing	30		14		8		6		
90-day mortality	69	(4)	40	(4)	12	(3)	7	(5)	0.223
Adjuvant therapy									0.009
No	724	(43)	371	(59)	200	(32)	62	(10)	
Chemotherapy	783	(46)	429	(64)	185	(27)	61	(9)	
Radiotherapy	5	(<1)	2	(50)	0	(0)	2	(50)	
Chemoradiation	189	(11)	93	(53)	66	(38)	15	(9)	

Baseline characteristics of the surgery group data are presented in Table 2. The mean age was 73.5 years, most of the patients were male (*n* = 1253, 60%), and most were clinically staged as having cT1–2 (*n* = 486, 67%) and cN0 (*n* = 1326, 77%) disease. The majority underwent a distal gastrectomy (*n* = 1435, 69%) and an open approach (*n* = 1908, 94%). A curative gastrectomy was performed in 1946 patients (94%), and a palliative resection (pT4b or pM1 intraoperatively) was performed in 120 patients (6%).

**Table 2:** Baseline, surgical and histopathologic characteristics of 2077 patients treated with primary gastrectomy for cancer

Characteristic	All (*N* = 2077)		<5 weeks (*n* = 772)		5–8 weeks (*n* = 727)		>8 weeks (*n* = 578)		*p*
	*n*	(%)	*n*	(%)	*n*	(%)	*n*	(%)	
Baseline characteristics									
Age, year, mean (±SD)	73.5	(±10.1)	73.2	(10.4)	74.6	(8.9)	72.3	(11.1)	<0.001
Gender									0.345
Male	1253	(60)	461	(37)	429	(34)	363	(29)	
Female	824	(40)	311	(38)	298	(36)	215	(26)	
Malignancy history									0.393
No	1745	(84)	659	(38)	608	(35)	478	(27)	
Yes	332	(16)	113	(34)	119	(36)	100	(30)	
Tumor differentiation									0.008
Good to moderate	582	(34)	212	(31)	197	(33)	173	(40)	
Poor to undifferentiated	1131	(66)	467	(69)	404	(67)	260	(60)	
Missing	364		93		126		145		
cT stage									<0.001
T1	139	(19)	24	(17)	37	(27)	78	(56)	
T2	347	(48)	120	(35)	133	(38)	94	(27)	
T3	150	(21)	57	(38)	50	(33)	43	(29)	
T4a	84	(12)	45	(54)	24	(29)	15	(18)	
Tx	1357		526		483		348		
cN stage									<0.001
N0	1326	(77)	440	(33)	483	(36)	403	(30)	
N+	387	(23)	181	(47)	136	(35)	70	(18)	
Nx	364		151		108		105		
Year of diagnosis									<0.001
2006–2008	934	(45)	426	(46)	308	(33)	200	(21)	
2009–2011	608	(29)	187	(31)	220	(36)	201	(33)	
2012–2014	535	(26)	159	(30)	199	(37)	177	(33)	
Referral status									<0.001
Diagnosis in treatment hospital	1657	(86)	674	(41)	585	(35)	398	(24)	
Diagnosis in other hospital	262	(14)	60	(23)	81	(31)	121	(46)	
Missing	158		38		61		59		
Treatment and histopathologic characteristics									
Surgical type									0.008
Distal gastrectomy	1435	(69)	545	(38)	501	(35)	389	(27)	
Total gastrectomy	607	(29)	206	(34)	215	(35)	186	(31)	
Multiorgan surgery	35	(2)	21	(60)	11	(31)	3	(9)	
Surgical approach									0.002
Open	1908	(94)	728	(38)	671	(35)	509	(27)	
Laparoscopic	129	(6)	33	(26)	45	(35)	51	(40)	
Missing	40		11		11		18		
Radicality									0.003
R0	1689	(85)	595	(35)	599	(35)	495	(29)	
R1–2	291	(15)	126	(43)	106	(36)	59	(20)	
Rx	97		51		22		24		
pT stage									<0.001
T1	454	(22)	89	(20)	156	(34)	209	(46)	
T2	321	(16)	104	(32)	121	(38)	96	(30)	
T3	747	(36)	321	(43)	259	(35)	167	(22)	
T4a	489	(24)	234	(48)	166	(34)	89	(18)	
T4b	55	(3)	23	(42)	21	(38)	11	(20)	
Tx	11		1		4		6		<0.001
pN stage									
N0	995	(48)	291	(29)	346	(35)	358	(36)	
N1	347	(17)	137	(39)	127	(37)	83	(24)	
N2	336	(16)	146	(43)	121	(36)	69	(21)	
N3	390	(19)	193	(49)	130	(33)	67	(17)	
Nx	9		5		3		1		
pM stage									0.162
M0	2003	(96)	737	(37)	707	(35)	559	(28)	
M1	74	(4)	35	(47)	20	(27)	19	(26)	
Curative intent									0.035
Yes	1946	(94)	713	(37)	687	(35)	546	(28)	
No	120	(6)	58	(48)	36	(30)	26	(22)	
Missing	11		1		4		6		
90-day mortality	240	(12)	94	(12)	68	(9)	78	(13)	0.053
Adjuvant therapy									0.043
No	1993	(96)	730	(37)	704	(35)	559	(28)	
Chemotherapy	28	(1)	11	(39)	6	(21)	11	(39)	
Radiotherapy	1	(<1)	1	(100)	0	(0)	0	(0)	
Chemoradiation	55	(3)	30	(55)	17	(31)	8	(15)	

### WT and Related Variables

The WT to neoadjuvant therapy was missing in 215 patients (13%), the result of an unknown start date for neoadjuvant treatment. Median WT was 4.6 weeks (interquartile range [IQR] 3.4–6.0). The median WT did not significantly differ between the curative (pT1–4a, N0–3, M0) and palliative (pT4b and/or pM1) treated patients (4.6 vs. 4.1 weeks, *p* = 0.136). The groups of ≤5 weeks, 5–8 weeks, and >8 weeks consisted of 895 (60%), 451 (30%), and 140 (9%) patients, respectively (Table 1). The only difference with regard to baseline characteristics included referral status (*p* < 0.001). Surgical and histopathologic characteristics differed on ypT stage (*p* < 0.001), ypN stage (*p* = 0.036), and adjuvant therapy (*p* = 0.009). Patients with pathologic advanced tumor stages had significantly shorter WTs compared to patients with early tumor stages.

In the surgery group, the WT was available in all 2077 patients. Median WT was 6.0 weeks (IQR 4.3–8.4). The median WT was significantly longer in the curative-treated patients compared to the palliative-treated patients (6.1 vs. 5.1 weeks, *p* = 0.005). The groups of ≤5 weeks, 5–8 weeks, and >8 weeks consisted of 772 (37%), 727 (35%), and 578 (28%) patients, respectively (Table 2). Age (*p* < 0.001), tumor differentiation (*p* = 0.008), cT stage (*p* < 0.001), cN stage (*p* < 0.001), year of diagnosis (*p* < 0.001), and referral status (*p* < 0.001) all significantly differed between the groups. Most importantly, patients with clinical advanced tumor stages had a significantly shorter WT to gastrectomy compared to patients with early tumor stages. Furthermore, WT increased over the years both in the categorized WT groups (Table 2) as in median WT (2006–2008: 5.4 weeks; 2009–2011: 6.6 weeks; 2012–2014: 6.7 weeks). Regarding surgical and histopathologic characteristics, surgical type (*p* = 0.008), surgical approach (*p* = 0.002), radicality (*p* = 0.003), pT stage (*p* < 0.001), pN stage (*p* < 0.001), curative intent (*p* = 0.035), and adjuvant therapy (*p* = 0.043) differed significantly between the WT groups. Patients who underwent an irradical resection or a resection for pathologic advanced tumor stages had significantly shorter WTs.

### Overall Survival

Median OS in the neoadjuvant group was 32 months (range 1–118 months), and 1-, 3-, and 5-year survival rates were 86, 58, and 47%, respectively. The categorized and linear effect of WT on OS is presented in Table 3. In multivariable analysis, both the categorized and the discrete variables of WT were not identified as independent prognostic factors associated with OS (WT 5–8 weeks vs. ≤5 weeks, HR 0.82, *p* = 0.68; >8 weeks vs. ≤5 weeks, HR 0.85, *p* = 0.354; and additional week WT, HR 0.96, *p* = 0.065).

**Table 3:** Univariable and multivariable Cox proportional hazard analysis on influence of WT on risk of death in patients treated with neoadjuvant chemotherapy followed by gastrectomy or primary gastrectomy for cancer

Characteristic	Univariable		Multivariable^a^	
	HR (95% CI)	*p*	HR (95% CI)	*p*
Neoadjuvant treatment				
WT ≤5 weeks	Ref	–	Ref	–
WT 5–8 weeks	0.79 (0.67–0.94)	0.008*	0.82 (0.66–1.02)	0.068
WT >8 weeks	0.80 (0.62–1.05)	0.106	0.85 (0.59–1.21)	0.354
Additional week WT	0.97 (0.94–1.01)	0.057	0.96 (0.92–1.00)	0.078
Surgery only				
WT ≤5 weeks	Ref	–	Ref	–
WT 5–8 weeks	0.86 (0.76–0.98)	0.018*	0.92 (0.81–1.05)	0.200
WT >8 weeks	0.75 (0.65–0.86)	<0.001*	0.95 (0.79–1.08)	0.314
Additional week WT	0.97 (0.96–0.99)	<0.001*	0.99 (0.98–1.01)	0.264

*CI* confidence interval, *HR* hazard ratio, *WT* waiting time

* Statistically significant (*p* < 0.05)

^a^Adjusted for baseline characteristics: age, gender, malignancy history, tumor differentiation, cT stage, cN stage, year of diagnosis, and referral status

Median OS in the surgery group was 25 months (range 0–120 months), and 1-, 3-, and 5-year survival rates were 73, 46, and 37%, respectively. The categorized and linear effect of WT on OS is presented in Table 3. In multivariable analyses, both the categorized and discrete variables of WT were not identified as independent prognostic factors associated with OS (WT 5–8 weeks vs. ≤5 weeks, HR 0.91, *p* = 0.175; >8 weeks vs. ≤5 weeks, HR 0.92, *p* = 0.314; and additional week WT, HR 0.99, *p* = 0.264). Sensitivity analyses did not significantly change the HR estimates of the original multivariable analyses.

## Discussion

This population-based cohort study examined whether WT between diagnosis and start of treatment with curative intent was associated with OS in patients with gastric cancer. The results demonstrated that WT was not associated with OS in either patients treated with neoadjuvant chemotherapy or primary gastrectomy. The study is novel in that it is the first to be conducted in the West and the first to evaluate the association of WT between diagnosis and the start of neoadjuvant therapy, which has become the standard of care.

This study was conducted with the hypothesis that longer WTs might lead to a worse OS through disease progression, as has been shown for breast cancer, uterine cancer, and head and neck cancer.^17^–^19^ For lung cancer, pancreatic head cancer, colorectal cancer, and esophageal cancer, however, studies could not demonstrate this relationship.^8^,^14^,^20^–^22^ For gastric cancer specifically, a previous study reported that a longer WT did not adversely affect survival.^11^ Unfortunately, this study did not investigate the WT between diagnosis and the start of neoadjuvant treatment, and it was conducted in an Asian population, which differs significantly from a Western population.^12^ The results of the present study are therefore reassuring and confirm that WT does not adversely affect survival. The varying impact of WT for different cancer types may be explained by differences in duration of patient delay and diagnostic delay. The variety of these delays between cancer types may be related to the cancer site, time of manifestation of symptoms, and presence or absence of screening programs. Compared to these delays (months to years), WT from diagnosis to treatment (weeks to months) is relatively short and may have a negligible effect on OS, as this study demonstrated for gastric cancer.^23^

Interestingly, this study found that in both groups, patients with pathologic advanced tumor stages had shorter WTs than patients diagnosed with pathologic early tumor stages. In the surgery group, this difference was already present at baseline, and therefore, it is assumed that patients with clinical advanced tumor stages may have had priority and may have been scheduled for surgery earlier. On the other hand, it can be hypothesized that some patients with clinical advanced tumor stages with a long WT may have developed disease progression as a result of this longer WT and eventually may have dropped out for surgery, and hence were not included in this study. Unfortunately, these dropouts could not be extracted from the NCR. Because both dropouts and palliative gastrectomies are due to disease progression, it is unlikely that there is a significant amount of dropouts with longer WTs. Because this study did not show a clinically relevant difference in WT between curative resections and palliative resections, we assume that dropouts were not likely to have affected the results significantly. In the neoadjuvant group, it remains unclear why patients with pathologic advanced tumor stages had shorter WTs. Early treatment due to worse tumor characteristics seems unlikely, as cTN stages were comparable between the WT groups, and moreover, no waiting list exists for the start of neoadjuvant chemotherapy in the Netherlands. Although median WTs in the palliative group did not exceed the median WTs in the curative group (both around 4 weeks), longer WTs might have caused dropouts, which resulted in patient selection. Furthermore, a discrepancy in clinical and pathologic tumor stages should be interpreted with caution for two reasons. First, imaging strategies currently used for clinical TN staging all have their specific limitations, and accurate prediction of pathologic TN stage is still not possible for an individual patient.^24^ Second, neoadjuvant chemotherapy downsizes the tumor and influences pathologic tumor stages.

In more recent years, WT to primary surgery increased, whereas WT to neoadjuvant therapy remained stable. We assume that this can be explained by the fact that most patients who underwent primary surgery after 2008 were not eligible for perioperative chemotherapy—for instance, as a result of poor condition. It is expected that in these patients, more time was needed to optimize them before the start of treatment. Moreover, patients treated with primary surgery needed more referral in the recent years (data not shown). Because additional time is needed for referral, the WT may have increased in this group.

In the Netherlands, WT is seen as an important quality indicator by several authorities.^9^,^10^ This study demonstrated that 57% of the patients who underwent neoadjuvant treatment, and 37% of the patients who underwent primary surgery, comply with this recommendation. For gastric cancer, this study demonstrated that there is no absolute need to strictly adhere to this recommendation for oncologic reasons. Factors such as patient delay and delay to diagnosis are probably more relevant for oncologic outcomes, as these time frames are relatively longer than the WT from diagnosis to treatment.^23^ Because WT does not impair oncologic outcomes within a clinically relevant time frame, it may be used to optimize these often malnourished patients before the start of the intensive treatment.^25^ In fact, optimizing patients could be an explanation for the fact that many patients were not treated within 5 weeks in our cohort. On the other hand, WT can have a negative (psychosocial) influence on patient factors such as quality of life.^7^ Efforts should therefore be made to keep the WT to a minimum. During WT, the results from this study can be used to reduce psychosocial distress by reassuring patients that longer WTs do not adversely affect oncologic outcomes.

This study has some limitations. First, although we adjusted for most of the generally accepted baseline characteristics in gastric cancer, other potential confounding factors may have been missed. For instance, data on patients’ comorbidities, performance status, or nutritional status, symptoms of the tumor, and hospital factors were not available from the NCR database, but these data might have influenced the results. Second, although data from the NCR are being directly extracted from medical records within 9 months after diagnosis, the study was retrospective in nature, and possible confounders may have been missed. On the other hand, a randomized trial would not be ethical to evaluate the influence of WTs. Therefore, multivariable analysis as performed in this study will remain one of the best methods to investigate this topic. Nonetheless, this is the first nationwide cohort study investigating the effect of WT on survival in patients with gastric cancer in the West. The results of this study can be extrapolated to other countries in the West because treatment and outcomes of gastric cancer care are comparable and therefore can be used for the composition of (international) guidelines on gastric cancer treatment.^26^

In conclusion, this large population-based cohort study demonstrated that a longer WT between diagnosis and start of treatment with curative intent for gastric cancer is not associated with worse OS. Although efforts should be made to keep WT to a minimum for patients’ quality of life, these results could help establish new cancer-specific guidelines and reassure patients with gastric cancer.
